# Effect of Long-Term Exercise on Liver Lipid Metabolism in Chinese Patients With NAFLD: A Systematic Review and Meta-Analysis

**DOI:** 10.3389/fphys.2021.748517

**Published:** 2021-11-22

**Authors:** Ye Gao, Jiandong Lu, Xinhong Liu, Jingqi Liu, Qirui Ma, Yajun Shi, Hao Su

**Affiliations:** ^1^The Graduate School, Beijing Sport University, Beijing, China; ^2^The School of Sports Science, Beijing Sport University, Beijing, China

**Keywords:** long-term exercise, non-alcoholic fatty liver disease, lipid metabolism, meta-analysis, systematic review

## Abstract

**Purpose:** Using meta-analysis to evaluate the effect of various long-term exercises (more than 4 weeks) on liver lipid metabolism of Chinese patients with non-alcoholic fatty liver disease (NAFLD) and provides more targeted exercise recommendations.

**Methods:** Four databases consisting of PubMed, Web of Science, China Science and Technology Journal Database (VIP), China Knowledge Resource Integrated Database (CNKI) were searched up to May 2021. Randomized controlled trials (RCTs) were eligible, and the outcomes of body composition, lipid metabolism [including triglyceride (TG), total cholesterol (TC), low-density lipoprotein-cholesterol (LDL-C), and high-density lipoprotein-cholesterol (HDL-C)], and liver function [including alanine aminotransferase (ALT) and aspartate aminotransferase (AST)] were used to assess the effectiveness of long-term exercise on Chinese patients with NAFLD.

**Results:** Eleven articles with a total of 13 data points (involving 1,006 participants) satisfied the inclusion criteria and were pooled in the meta-analysis. The findings demonstrated that long-term exercise decreased the level of TG [−0.50, 95%CI (−0.64, −0.36)], TC [−0.55, 95%CI (−0.92, −0.18)], LDL-C [−0.29, 95%CI (−0.43, −0.15)], ALT [−3.45, 95%CI (−6.78, −0.12)], AST [−6.91, 95%CI (−10.00, −3.81)], and body mass index (BMI) of patients who did exercise last more than 6 months [−1.55, 95%CI (−2.32, −0.79)] significantly. The effect on HDL-C was not obvious.

**Conclusion:** Long-term exercise can improve the levels of TG, TC, LDL-C, ALT, and AST in Chinese patients with NAFLD significantly, and exercise last more than 6 months can decrease the BMI of Chinese patients with NAFLD.

## Introduction

Non-alcoholic fatty liver disease (NAFLD) is a metabolic-stress liver injury. Epidemiological studies showed that the worldwide prevalence of NAFLD is about 6.3%–45% (Younossi et al., 2016). With the escalating rates of obesity in China, the incidence of NAFLD is in the middle to the upper level (Younossi et al., 2016). Currently, NAFLD has become the principal cause of abnormal liver biochemical indicators in health checkups in China.

Metabolic disturbance of lipids is a hallmark of NAFLD, usually manifested as triglyceride (TG) accumulation in hepatocytes (Day and James, 1998, 2010), an increase of plasma TG, total cholesterol (TC), low-density lipoprotein-cholesterol (LDL-C), and decrease of high-density of lipoprotein-cholesterol (HDL-C) (DeFilippis et al., 2013; Peng et al., 2017), which may cause worse consequences, such as atherosclerosis or type 2 diabetes (Byrne and Targher, 2015). Therefore, improving the lipid metabolism of patients with NAFLD is essential for delaying their condition.

Until now, NAFLD lacks surgical options and pharmacological treatments with significant results. Thus, intervention for NAFLD using non-pharmacological means is becoming a hot topic. Exercise intervention as a considerable means of non-pharmacological intervention has shown to have a positive effect on NAFLD (Romero-Gomez et al., 2017; Farzanegi et al., 2019; Nath et al., 2020). Long-term exercise, defined as cumulative, structured, and planned, repetitive bouts of physical activity lasting ≥4 weeks (Caspersen et al., 1985; Wilmore et al., 2008), are more effective on lipid metabolism in patients with NAFLD than short-term exercise (Bilet et al., 2015), and although studies were chosen to analyze the effects of long-term exercise interventions on lipid metabolism in patients with NAFLD, the literature included in the existing meta-analysis is mainly in English, and its ethnic composition is significantly different from that in China, which makes the analysis of long-term exercise interventions in Chinese NAFLD patients not comprehensive. A comprehensive quantitative evaluation of the effectiveness of long-term exercise interventions on lipid metabolism in Chinese patients with NAFLD is lacking. Therefore, this study intends to select randomized controlled trials (RCTs) on long-term exercise in Chinese patients with NAFLD, to evaluate the consistency between the results of different studies using meta-analysis, and to evaluate the lipid metabolism intervention of various exercise programs in Chinese patients with NAFLD at different age levels, to provide more targeted exercise recommendations for Chinese patients with NAFLD.

## Methods

### Search Strategy

Relevant research articles from the construction of the database to May 2021 were collected with keywords such as “exercise,” “training,” “liver,” from the following databases: PubMed, Web of Science, China Science and Technology Journal Database (VIP), China Knowledge Resource Integrated Database (CNKI). The complete search used for PubMed was: (exercise [MeSH Terms] OR training [MeSH terms]) AND liver [MeSH terms]. The search was imposed with the limitation of RCTs and without language or status limitations. Supplementary Material details all of the search strategies used in this study.

### Inclusion Criteria

The inclusion criteria followed the PICOS principle (i.e., population, intervention, comparison, outcome, and study design): (1) All NAFLD patients included in this study were definitively diagnosed by pathological examination. (2) Interventions need to include exercise interventions for at least 4 weeks. (3) The control group remained in the same condition as the study group, except for no exercise intervention. (4) Outcomes need to include: body mass index (BMI), TG, TC, LDL-C, HDL-C, alanine aminotransferase (ALT), and aspartate aminotransferase (AST). Mean and standard deviation (SD) were reported in the trials. (5) Only the trials designed as RCTs were covered.

### Exclusion Criteria

Trials were excluded when they met the exclusion criteria as the following: (1) Conference abstracts, case reports, comments, review, and experimental animal studies. (2) Outcomes did not meet inclusion criteria. (3) Mean and SD could not be obtained from the articles or authors.

### Studies Selection

Two reviewers (YG and XL) independently reviewed the titles or abstracts of all studies. The full contents of the relevant studies were checked carefully to evaluate whether the study could be included. Any disagreements were resolved by discussion or consultation with a third independent reviewer (QM) if necessary.

### Quality Assessment

The Cochrane Collaboration tool was used to evaluate the risk of bias of the included trials (Higgins et al., 2011). Two reviewers (YG and XL) independently evaluate seven domain biases as follows: random sequence generation (selection bias), allocation concealment (selection bias), blinding of participants and personnel (performance bias), blinding of outcome assessment (detection bias), incomplete outcome data (attrition bias), and selective reporting (reporting bias). Three grades of high, low, or unclear bias were labeled for every study included. Disagreements were resolved by discussion or consulting with a third independent reviewer (QM) if necessary.

### Data Extraction

The two reviewers independently extracted the data from every included eligible trial as the following: study characteristics (i.e., author and year), participant characteristics (i.e., age and number of participants), description of interventions, and outcomes. Any disagreements were settled by discussion to reach unanimity, and the authors of the trials were contacted directly to acquire original studies and data if necessary.

### Statistical Analysis

All statistical analyses were performed using R (RStudio V1.4.1106, Boston, Massachusetts, USA), with the meta package. The percentage of variation across studies indicative of heterogeneity was reported using the *I*^2^ statistic and the Chi^2^ test. Interpretation of the *I*^2^ statistic was in accordance with Cochrane guidelines as following: <40% might not be important; 30–60% may represent moderate heterogeneity; 50–90% may represent substantial heterogeneity; and >75% considerable heterogeneity (Higgins et al., 2021). A statistically significant effect based on the Chi^2^ test suggested evidence of heterogeneity. If *p* < 0.05, it was considered to be a significant difference. The presence of moderate and substantial heterogeneity may provoke further investigation through a subgroup analysis of moderator variables (duration of intervention, age groups, and frequencies). Sensitivity analysis was used to study the source of heterogeneity and evaluate the stability of the results by removing each trial one by one. All experimental data were continuous variables. The value of quantitative data was shown by the mean difference (MD) with 95% confidence interval (CI). The possible publication bias was evaluated by Funnel plot asymmetry and Begg's test if more than nine trials were included.

## Results

### Search Results

**Figure 1:**
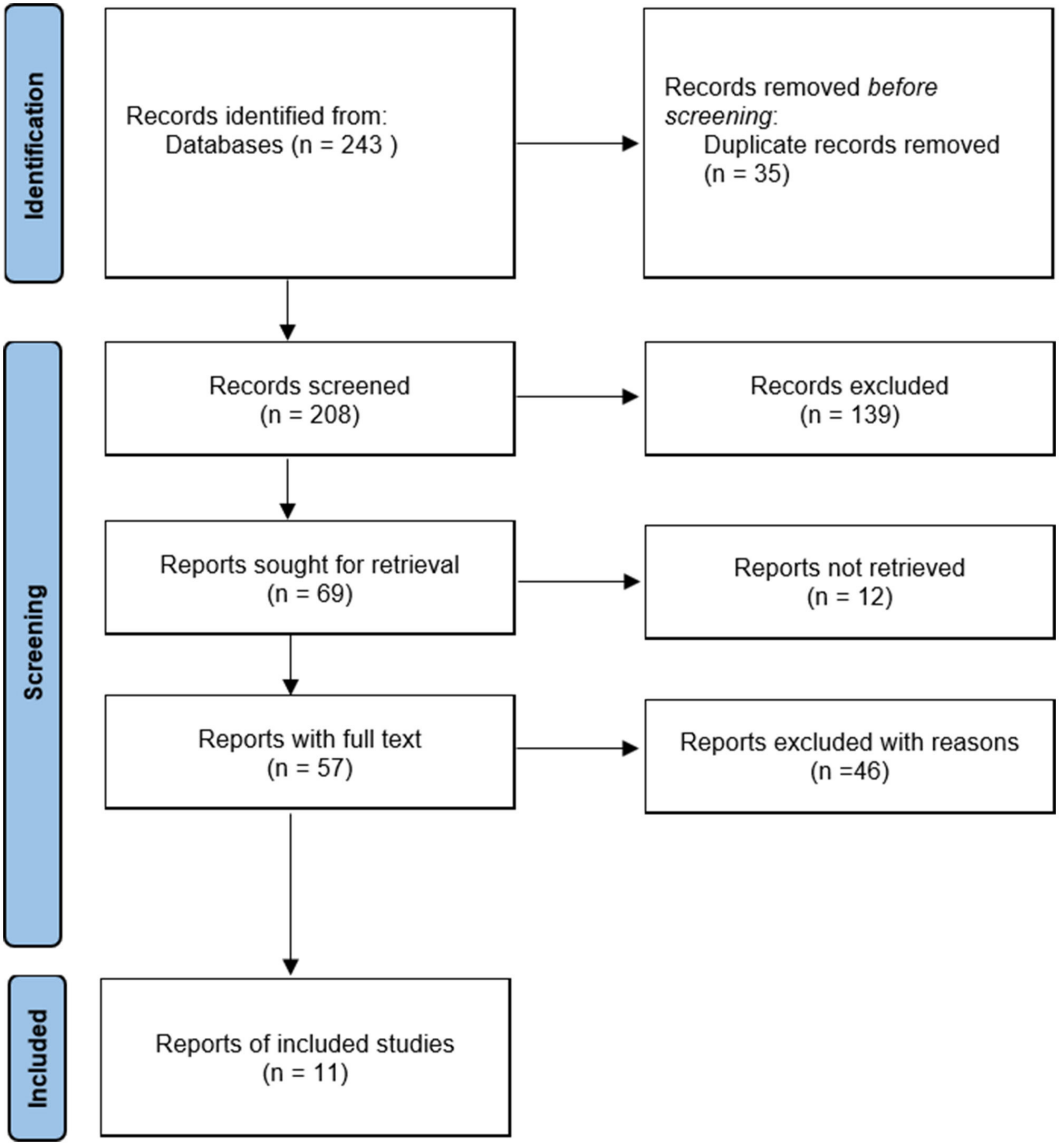
Flow diagram of the study selection.

From our initial search, 243 records were obtained. After reviewing the information on the articles' titles and abstracts, 69 potentially eligible articles were identified. After reviewing the full content of the studies, 11 articles with 13 data points (1,006 participants) satisfied the inclusion criteria and were pooled in this meta-analysis (Yang, 2006; Wang, 2008; Chen and Liu, 2011; Feng et al., 2013; Wu and Gao, 2014; Yan and Lu, 2014; Yao et al., 2018; Zuo et al., 2019; Liu et al., 2020; Luo et al., 2020; Peng et al., 2021). The details of the process of identifying articles from initially searching to inclusion are shown in Figure 1.

### Description of Included Studies

The characteristics of the included articles were summarized in Table 1, including the name of the first author, publication year, groups, participants, gender, age, intervention methods. One article was published in English, and ten in Chinese.

**Table 1:** Characteristics of included studies.

**Author, Year**	**Groups**	**Participants**	**Gender (M/F)**	**Age**	**Exercise type**	**Duration (week)**	**Frequency (*n*/week)**	**Intensity**	**Duration (min)**	**Outcomes**
Luo et al., 2020	Exercise	26	No mention	29.69 ± 7.77	HIIT	12	3	55–70% HRmax	60	➁➂➃➄➅➆
	Control	25		30.96 ± 7.15	No exercise					
Yao et al., 2018	Exercise 1	29	7/22	61.28 ± 7.52	Aerobic	22	3	60–70% HRmax	60	➀➁➂➅
	Exercise 2	31	16/15	55.80 ± 12.29	Resistance			60–70% 1RM		
	Control	31	13/18	58.06 ± 9.79	No exercise					
Feng et al., 2013	Exercise	60	35/25	30 ± 2	Aerobic	24	3	60%VO2max	40–60	➀➁➂➅
	Control	60	34/26	30 ± 3	No exercise					
Zuo et al., 2019	Exercise	12	0/24	46.5 ± 4.1	Aerobic	24	4	60–75% HRmax	60	➀➁➂➃➄
	Control	12			No exercise					
Liu et al., 2020	Exercise 1	49	35/14	41.14 ± 11.55	Aerobic	12	5	Resting heart rate + 40–60% Reserve heart rate	30–60	➀➂➃➄
	Exercise 2	49	40/9	39.82 ± 9.97	Aerobic					
	Control	49	38/11	44.84 ± 10.20	No exercise					
Yang, 2006	Exercise	132	No mention	32–46	Aerobic	12	5	Tight breathing	60	➁➂➃➄
	Control	125			No exercise					
Wang, 2008	Exercise	30	60/0	52.6	Aerobic	48	3	Heart rate = (180-Age)	60	➀➁➂➃➄
	Control	30			No exercise					
Chen and Liu, 2011	Exercise	40	No mention	40.3 ± 6.8	Aerobic	8	3	60–70% HRmax	40–50	➁➂➃➄
	Control	40			No exercise					
Yan and Lu, 2014	Exercise	46	30/16	46 ± 6.5	Aerobic	24–32	4–6	60–70% HRmax	40–90	➁➂➃➄➅➆
	Control	46	31/15	45 ± 7.5	No exercise					
Wu and Gao, 2014	Exercise	15	No mention	54.3 ± 12.4	Aerobic	16	5	Resting heart rate + 40–60% reserve heart rate	60	➀➁➅
	Control	15		55.2 ± 12.7	No exercise					
Peng et al., 2021	Exercise	27	No mention	21.3 ±1.0	Aerobic	12	4	FATmax	90	➀➁➂➅➆
	Control	27		21.8 ± 0.8	No exercise					

*➀, BMI; ➁, TG; ➂, TC; ➃, LDL-C; ➄, HDL-C; ➅, ALT; ➆, AST*.

### Risk of Bias of Included Studies

**Figure 2:**
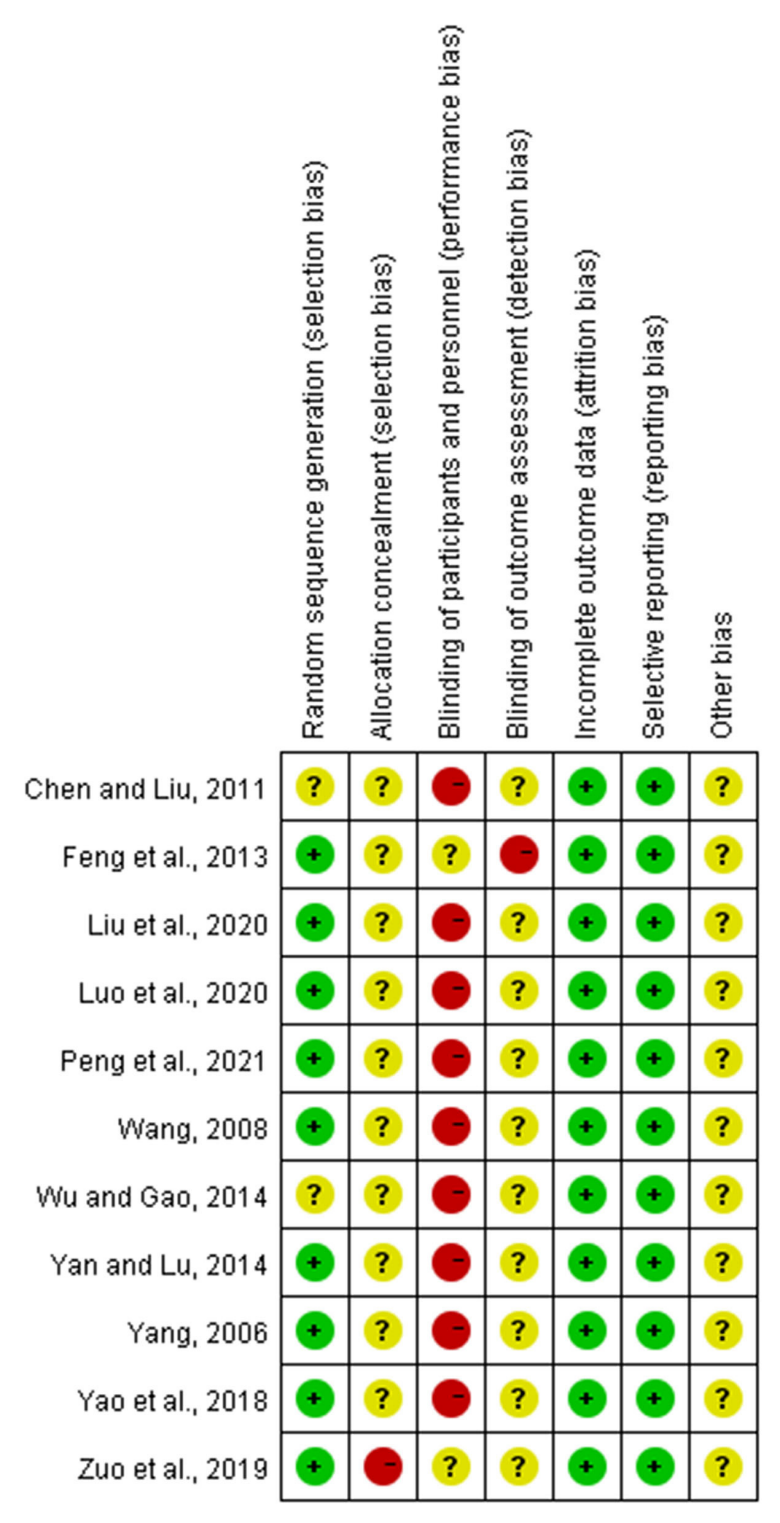
Risk of bias summary of included studies.

**Figure 3:**
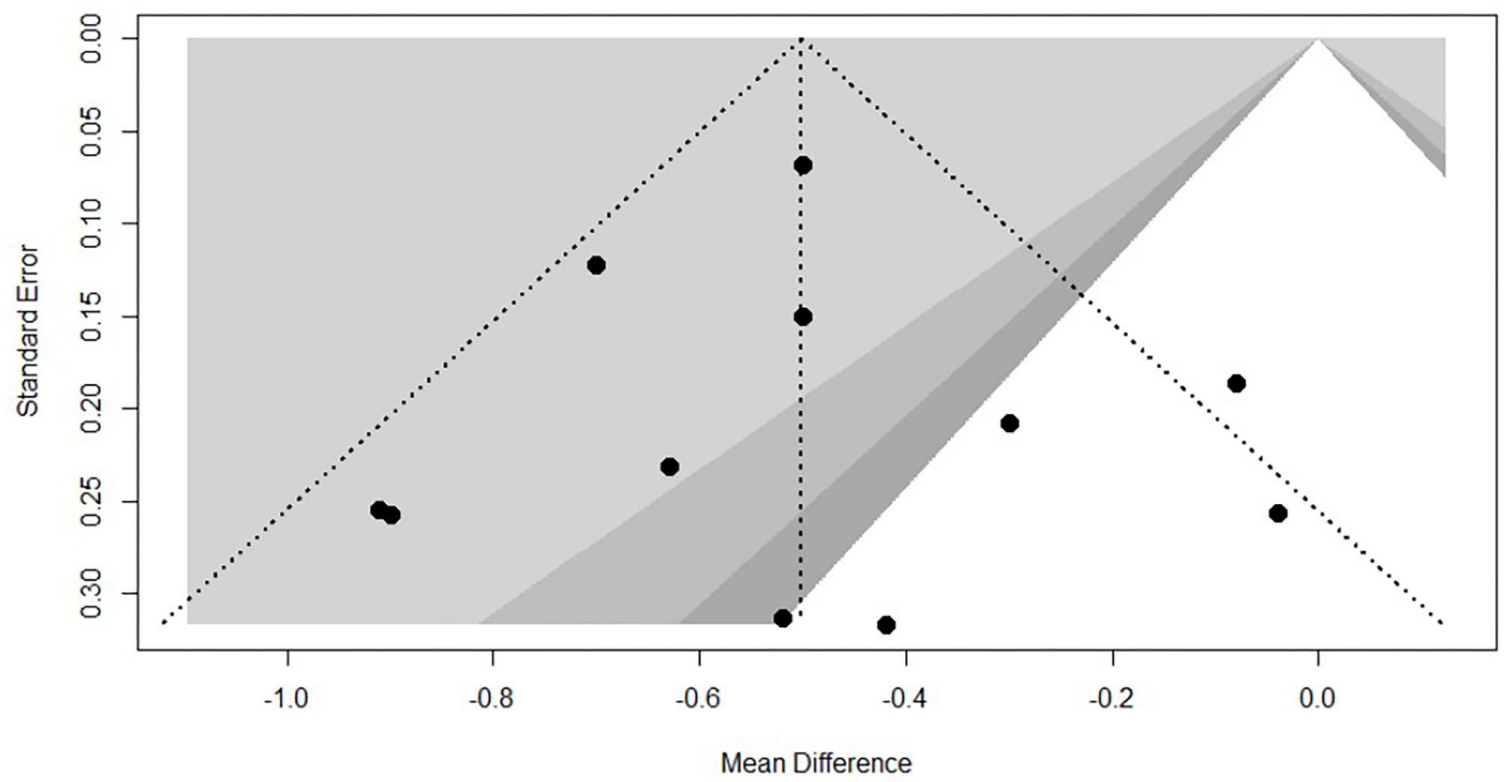
Funnel plot of TG.

**Figure 4:**
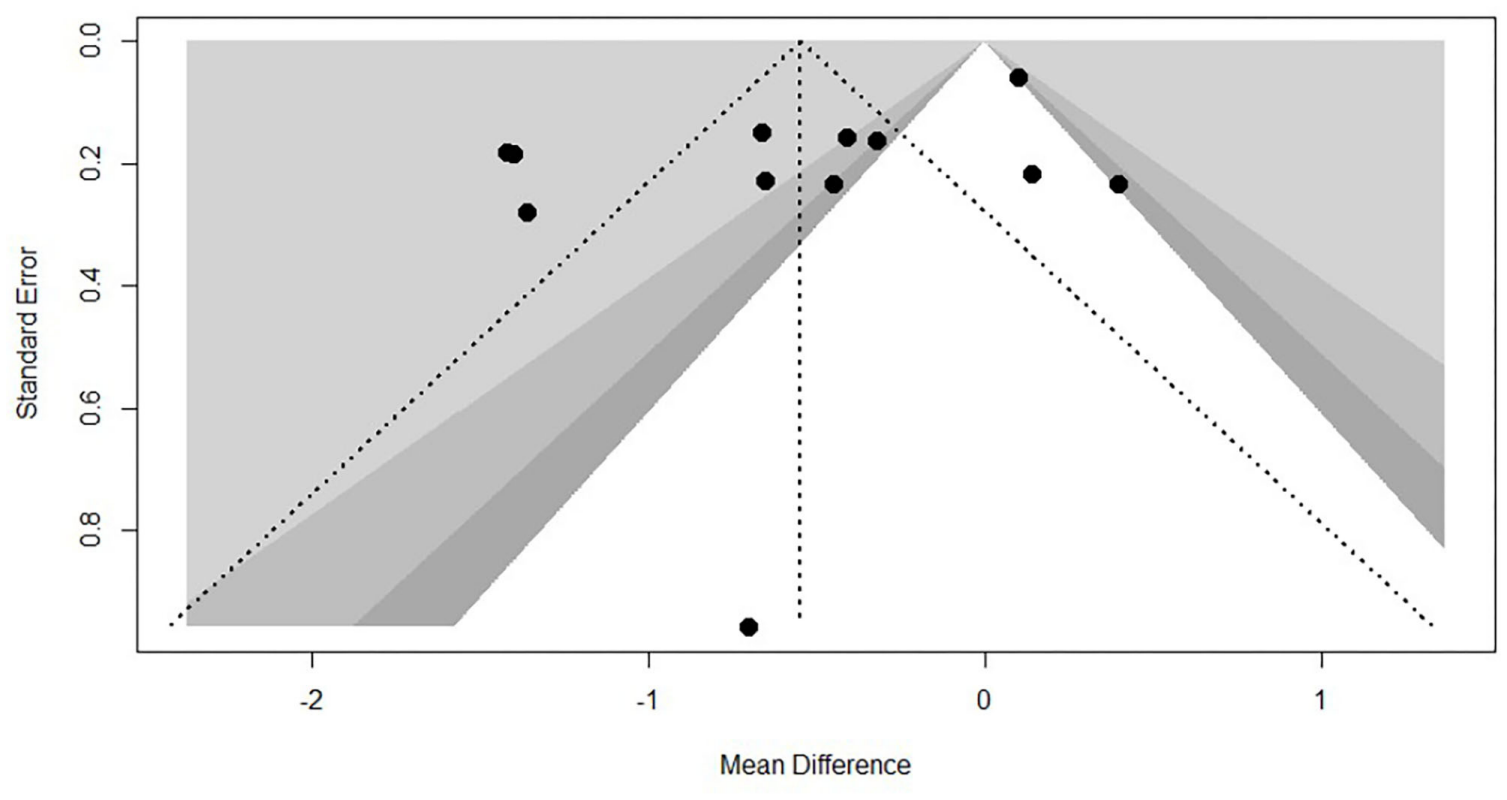
Funnel plot of TC.

Every included study was assessed for the risk of bias according to instructions by Higgins and Green (2011). As shown in Figure 2. Most (nine articles) of the studies used a randomization method, but none of these reported any information about allocation concealment. None of the trials met the requirements for the blinding of participants. However, it seems unfeasible to use the blinding method in view of the exercise intervention. No studies masked their long-term exercise intervention, which increased the risk of detection bias. All studies showed a low risk of incomplete outcome bias and selective reporting bias. It was unclear if the studies have additional bias. Funnel plot of TC and TG, showed in Figures 3, 4, presents the MD of each individual study against its own precision (standard error). Visual inspection of the funnel plot did not reveal significant asymmetry. The statistical testing of publication bias using Begg's regression did not reach statistical significance (Begg's test for TG, *p* = 0.88; Begg's test for TC, *p* = 1).

### Effects of Long-Term Exercise on BMI of Chinese Patient With NAFLD

**Figure 5:**
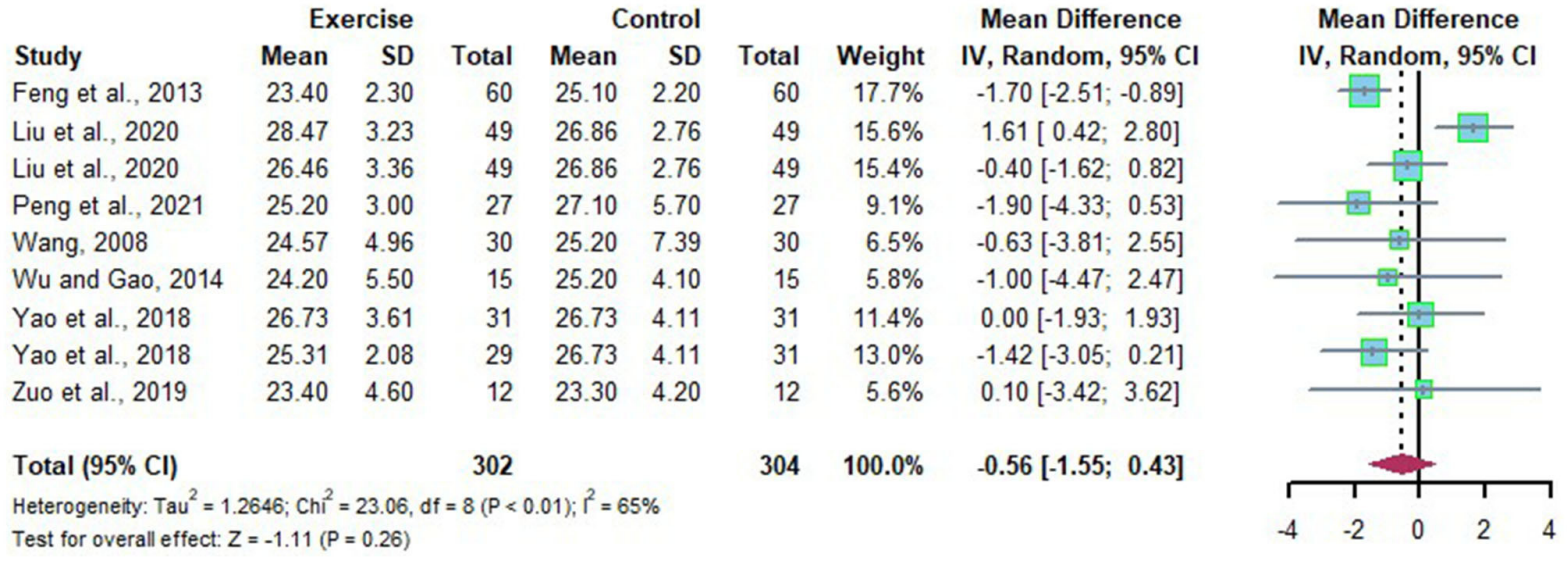
Meta-analysis of effects of long-term exercise on BMI of Chinese patient with NAFLD.

Nine data points reported the change of BMI in Chinese patients with NAFLD. Substantial heterogeneity was detected among the studies (*I*^2^ = 65%, *p* < 0.01). The random effects model revealed that the BMI in Chinese patients with NAFLD was not significantly reduced after long-term exercise intervention compared to that of the control group (*MD* = −0.56, 95%CI: −1.55 to 0.43, *P* = 0.26) (Figure 5). Subgroup analysis showed that long-term exercise lasts more than 6 months could significantly reduce BMI in Chinese Patients with NAFLD (*MD* = −1.55, 95%CI: −2.32 to −0.79, *p* < 0.0001), and no heterogeneity was detected in long-term exercise lasts more than 6 months subgroups (*I*^2^ = 0, *p* = 0.52) (Table 2).

**Table 2:** Subgroup analysis on BMI of Chinese patient with NAFLD.

	**Subgroup**	**Article**	**Data points**	***MD* [95% CI]**	***P***	***I*^2^ (%)**	***P_**heterogeneity**_***
Duration	<24 Weeks	Yao et al., 2018	6	−0.31 [−1.47, 0.84]	0.59	62	0.02
		Liu et al., 2020					
		Peng et al., 2021					
		Wu and Gao, 2014					
	≥24 Weeks	Feng et al., 2013	3	−1.55 [−2.32, −0.79]	<0.0001	0	0.52
		Zuo et al., 2019					
		Wang, 2008					
Frequency	3 Times/week	Yao et al., 2018	3	−0.80 [−1.96, 0.36]	0.18	0	0.54
		Wang, 2008					
	4 Times/week	Zuo et al., 2019	2	−1.26 [−3.26, 0.74]	0.22	0	0.36
		Peng et al., 2021					
	5 Times/week	Feng et al., 2013	4	−0.31 [−2.04, 1.42]	0.72	85	0.0001
		Liu et al., 2020					
		Wu and Gao, 2014					

Removing each trial one by one to evaluate the stability of the result, Liu's study was found to significantly affect the heterogeneity of included studies. The heterogeneity was reduced when this study was excluded (*MD* = −1.38, 95%CI: −2.00 to −0.76, *p* < 0.0001). Therefore, Liu's article was the key factor affecting the heterogeneity of studies.

### Effects of Long-Term Exercise on TG of Chinese Patient With NAFLD

**Figure 6:**
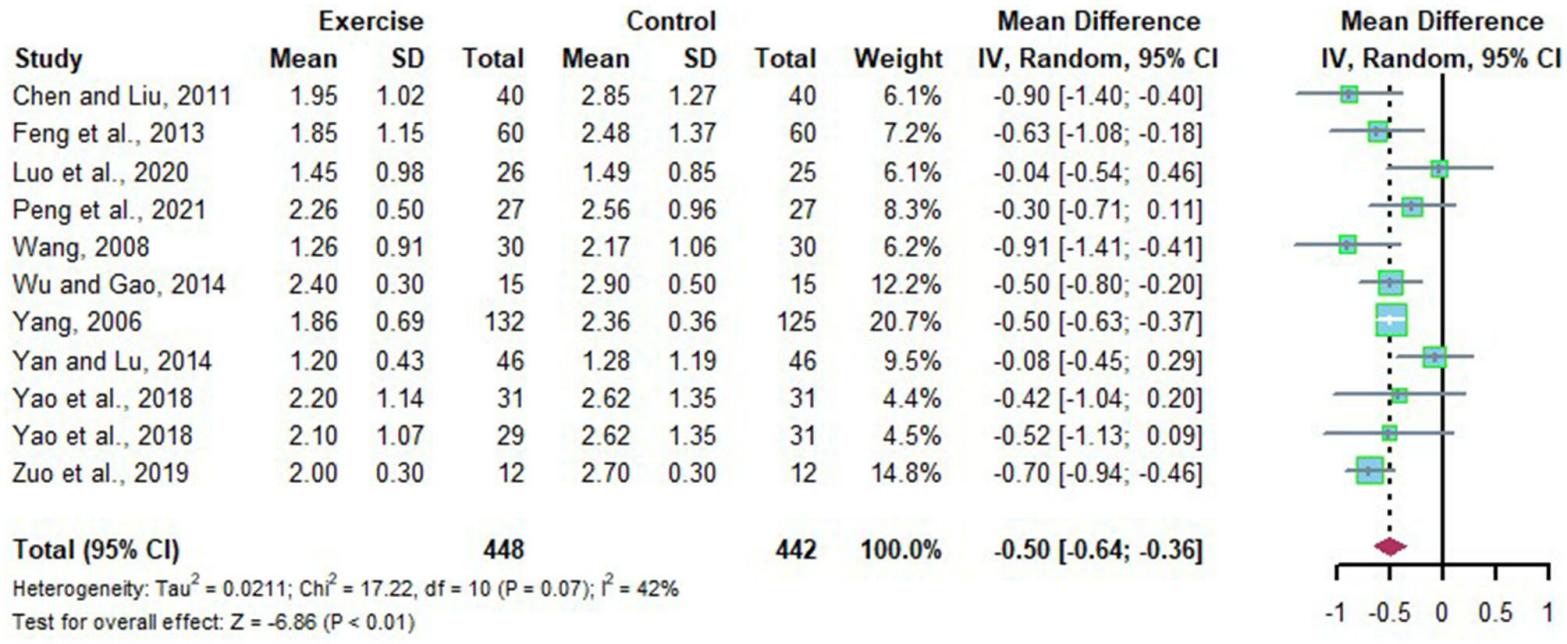
Meta-analysis of effects of long-term exercise on TG of Chinese patient with NAFLD.

Eleven studies revealed the change of TG in Chinese patients with NAFLD who received the long-term intervention. There were moderate heterogeneities detected among the studies (*I*^2^ = 42%, *p* = 0.07). The random effects model was applied for meta-analysis, and it revealed that the level of TG in Chinese patients with NAFLD was significantly decreased after long-term exercise intervention compared to that of the control group (*MD* = −0.50, 95%CI: −0.64 to −0.36, *p* < 0.01) (Figure 6).

### Effects of Long-Term Exercise on TC of Chinese Patient With NAFLD

**Figure 7:**
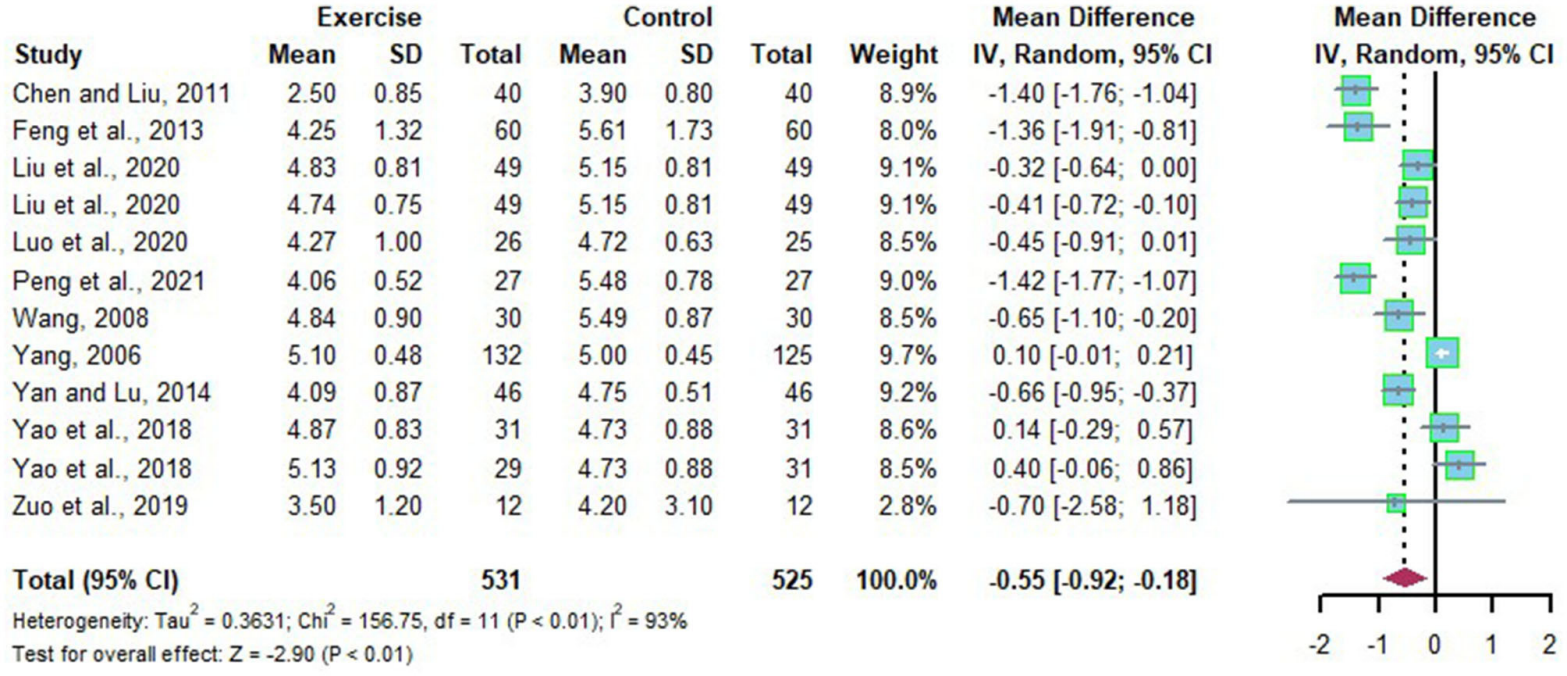
Meta-analysis of effects of long-term exercise on TC of Chinese patient with NAFLD.

A total of 12 data points explored the efficacy of long-term exercise intervention by evaluating the change of TC in Chinese patients with NAFLD with substantial heterogeneities (*I*^2^ = 93%, *p* < 0.01). Meta-analysis was performed with the application of the random effects model, and the source of heterogeneity was analyzed by subgroup analysis and sensitivity analysis. Compared to the control group, TC was significantly reduced after long-term exercise intervention in Chinese patients with NAFLD (*MD* = −0.55, 95%CI: −0.92 to −0.18, *p* < 0.01) (Figure 7). Subgroup analysis showed that long-term exercise intervention lasts more than 6 months or exercising four times a week could significantly reduce TC in Chinese patients with NAFLD (*MD* = −0.87, 95%CI: −1.20 to −0.55, *p* < 0.00001) (*MD* = −1.40, 95%CI: −1.74 to −1.05, *p* < 0.00001) (Table 3).

**Table 3:** Subgroup analysis on TC of Chinese patient with NAFLD.

	**Subgroup**	**Article**	**Data points**	***MD* [95% CI]**	***P***	***I*^2^ (%)**	***P_**heterogeneity**_***
Duration	<24 Weeks	Yao et al., 2018	8	−0.42 [−0.88, 0.03]	0.07	94	<0.00001
		Liu et al., 2020					
		Peng et al., 2021					
		Yang, 2006					
		Luo et al., 2020					
		Chen and Liu, 2011					
	≥24 Weeks	Yan and Lu, 2014	4	−0.87 [−1.20, −0.55]	<0.00001	38	0.18
		Feng et al., 2013					
		Zuo et al., 2019					
		Wang, 2008					
Age	≤ 30 Years old	Feng et al., 2013	3	−1.08 [−1.71, −0.45]	0.0008	83	0.003
		Peng et al., 2021					
		Luo et al., 2020					
	>30 Years old	Yao et al., 2018	9	−0.38 [−0.76, −0.00]	0.05	92	<0.00001
		Yan and Lu, 2014					
		Liu et al., 2020					
		Zuo et al., 2019					
		Yang, 2006					
		Wang, 2008					
		Chen and Liu, 2011					
Frequency	3 Times/week	Yao et al., 2018	5	−0.43 [−1.11, 0.25]	0.21	92	<0.00001
		Wang, 2008					
		Luo et al., 2020					
		Chen and Liu, 2011					
	4 Times/week	Zuo et al., 2019	2	−1.40 [−1.74, −1.05]	<0.00001	0	0.46
		Peng et al., 2021					
	5 Times/week	Yan and Lu, 2014	5	−0.49 [−0.93, −0.05]	0.03	92	<0.00001
		Feng et al., 2013					
		Liu et al., 2020					
		Yang, 2006					

Sensitivity analysis showed that outcomes of the effects of long-term exercise on TC of Chinese patients with NAFLD were stable when trials were removed one by one.

### Effects of Long-Term Exercise on LDL-C of Chinese Patient With NAFLD

**Figure 8:**
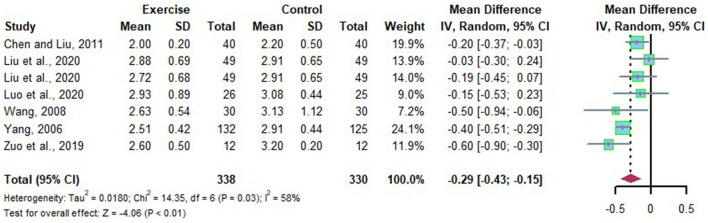
Meta-analysis of effects of long-term exercise on LDL-C of Chinese patient with NAFLD.

Seven studies evaluated the alteration of LDL-C in Chinese patients with NAFLD who received long-term exercise intervention. Moderate heterogeneities were detected among the included studies (*I*^2^ = 58%, *p* = 0.03). Subgroup analysis, sensitivity analysis was adopted to explore the source of heterogeneity. The random effects model was applied for meta-analysis, which suggests LDL-C was significantly declined after long-term exercise intervention compared to that of the control group in Chinese patients with NAFLD (*MD* = −0.29, 95%CI: −0.43 to −0.15, *p* < 0.01) (Figure 8). Subgroup analysis showed that long-term exercise lasts more than 6 months could significantly reduce LDL-C in Chinese patients with NAFLD (*MD* = −0.57, 95%CI: −0.82 to −0.32, *p* < 0.00001) with low heterogeneity (*I*^2^ = 0%, *p* = 0.72) (Table 4).

**Table 4:** Subgroup analysis on LDL-C of Chinese patient with NAFLD.

	**Subgroup**	**Article**	**Data points**	***MD* [95% CI]**	***P***	***I*^2^ (%)**	***P_**heterogeneity**_***
Duration	<24 Weeks	Liu et al., 2020	5	−0.20 [−0.38, −0.03]	0.04	60	0.04
		Yang, 2006					
		Luo et al., 2020					
		Chen and Liu, 2011					
	≥24 Weeks	Zuo et al., 2019	2	−0.57 [−0.82, −0.32]	<0.00001	0	0.72
		Wang, 2008					

The sensitivity analysis suggested that Yang's study had an impact on the heterogeneity. We found the heterogeneity was decreased when this study was removed (*MD* = −0.25, 95%CI: −0.41 to −0.09, *p* < 0.0001). Therefore, Yang's study was the key factor affecting the heterogeneity of studies.

### Effects of Long-Term Exercise on HDL-C of Chinese Patient With NAFLD

**Figure 9:**
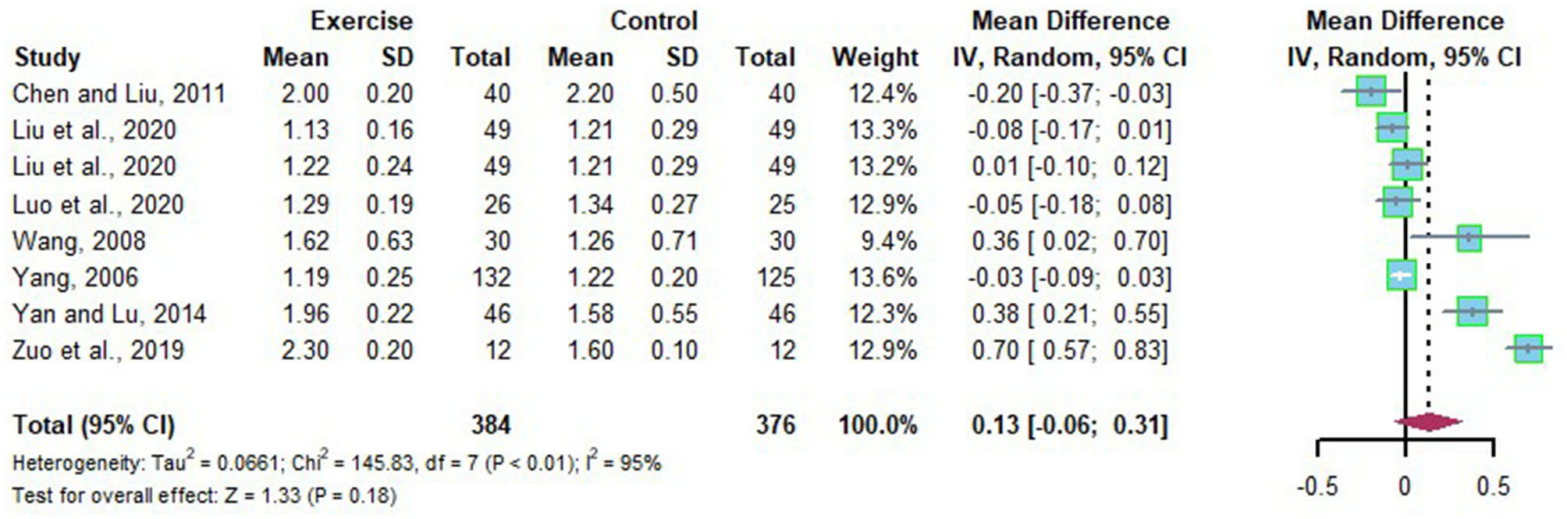
Meta-analysis of effects of long-term exercise on HDL-C of Chinese patient with NAFLD.

Eight studies reported the alteration of HDL-C in Chinese patients with NAFLD before and after long-term exercise intervention. Substantial heterogeneities were detected among the included studies (I^2^=95%, *p* < 0.01). The random effects model was applied for meta-analysis, and the source of heterogeneity was analyzed by subgroup analysis, sensitivity analysis. The random effects model revealed that HDL-C was not significantly increased after long-term exercise intervention compared to that of the control group in Chinese patients with NAFLD (*MD* = 0.13, 95%CI: −0.06 to 0.31, *p* = 0.18) (Figure 9). Subgroup analysis showed that long-term exercise more minor than half a year is not adequate for HDL-C (*MD* = −0.05, 95%CI: −0.08 to −0.01, *p* = 0.03), with low heterogeneity detected in long-term exercise less than half a year subgroups (*I*^2^ = 23%, *p* = 0.27) (Table 5).

**Table 5:** Subgroup analysis on HDL-C of Chinese patient with NAFLD.

	**Subgroup**	**Article**	**Data points**	***MD* [95% CI]**	***P***	***I*^2^ (%)**	***P_**heterogeneity**_***
Duration	<24 Weeks	Liu et al., 2020	5	−0.05 [−0.08, −0.01]	0.03	23	0.27
		Yang, 2006					
		Luo et al., 2020					
		Chen and Liu, 2011					
	≥24 Weeks	Yan and Lu, 2014	3	0.57 [0.47, 0.67]	<0.00001	80	0.006
		Zuo et al., 2019					
		Wang, 2008					

The source of heterogeneity was further explored by sensitivity analysis, which found that outcomes of the effects of long-term exercise on HDL-C of Chinese patients with NAFLD were stable when trials were removed one by one.

### Effects of Long-Term Exercise on ALT of Chinese Patient With NAFLD

**Figure 10:**
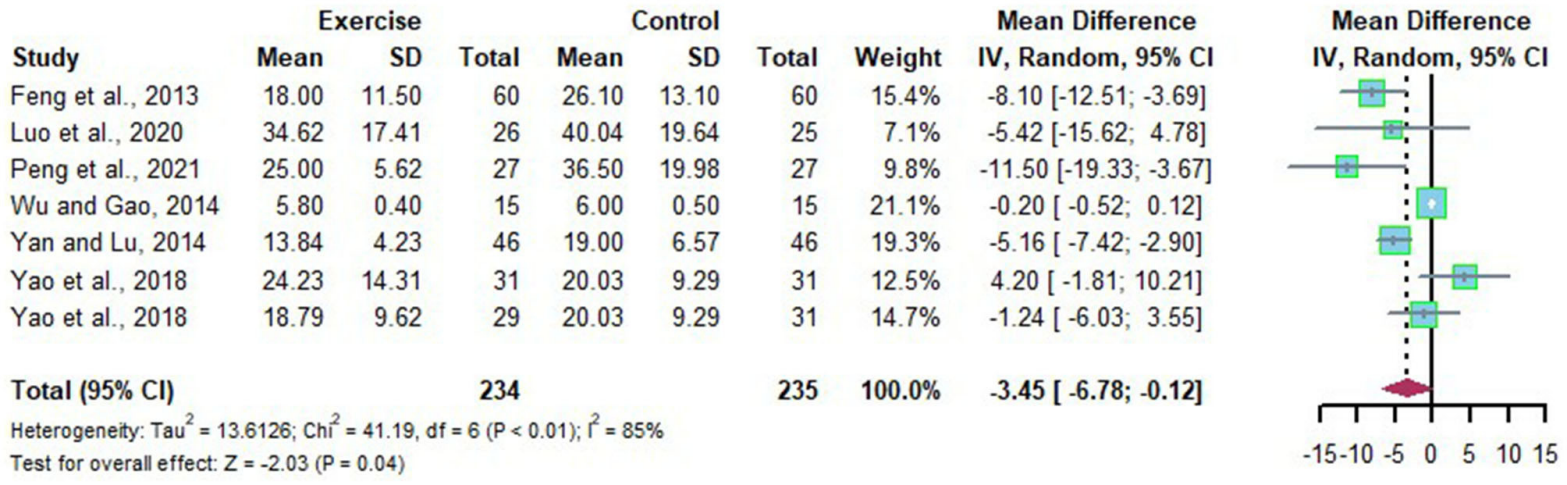
Meta-analysis of effects of long-term exercise on ALT of Chinese patient with NAFLD.

A total of seven studies assessed the efficacy of long-term exercise intervention in the treatment of NAFLD by analyzing the change of ALT with substantial heterogeneities (*I*^2^ = 85%, *p* < 0.01). Meta-analysis was conducted using the random effects model, and the source of heterogeneity was analyzed by subgroup and sensitivity analysis. The random effects model suggest that long-term exercise intervention tended to decrease ALT in Chinese patient with NAFLD significantly compared to that of the control group (*MD* = −3.45, 95%CI: −6.78 to −0.12, *p* = 0.04) (Figure 10). Subgroup analysis revealed that Chinese patients with NAFLD who exercised for more than or equal to 6 months at the time of the intervention or who were ≤ 30 years old were able to effectively reduce ALT levels after long-term exercise (*MD* = −5.77, 95%CI: −7.78 to −3.76, *p* < 0.00001), (*MD* = −8.48, 95%CI: −12.08 to −4.89, *p* < 0.00001) (Table 6).

**Table 6:** Subgroup analysis on ALT of Chinese patient with NAFLD.

	**Subgroup**	**Article**	**Data points**	***MD* [95% CI]**	***P***	***I*^2^ (%)**	***P_**heterogeneity**_***
Duration	<24 Weeks	Yao et al., 2018	5	−0.22 [−0.54, 0.11]	0.19	64	0.02
		Peng et al., 2021					
		Wu and Gao, 2014					
		Luo et al., 2020					
	≥24 Weeks	Yan and Lu, 2014	2	−5.77 [−7.78, −3.76]	<0.00001	26	0.24
		Feng et al., 2013					
Age	≤ 30 Years old	Feng et al., 2013	4	−8.48 [−12.08, −4.89]	<0.00001	0	0.62
		Peng et al., 2021					
		Luo et al., 2020					
	>30 Years old	Yao et al., 2018	3	−0.29 [−0.61, 0.03]	0.07	85	0.0001
		Yan and Lu, 2014					
		Wu and Gao, 2014					

The source of heterogeneity was searched for using an article-by-article exclusion, it was found that the exclusion of any article did not lead to a decrease in heterogeneity, indicating that the results of this part of the Meta-analysis were more robust.

### Effects of Long-Term Exercise on AST of Chinese Patient With NAFLD

**Figure 11:**
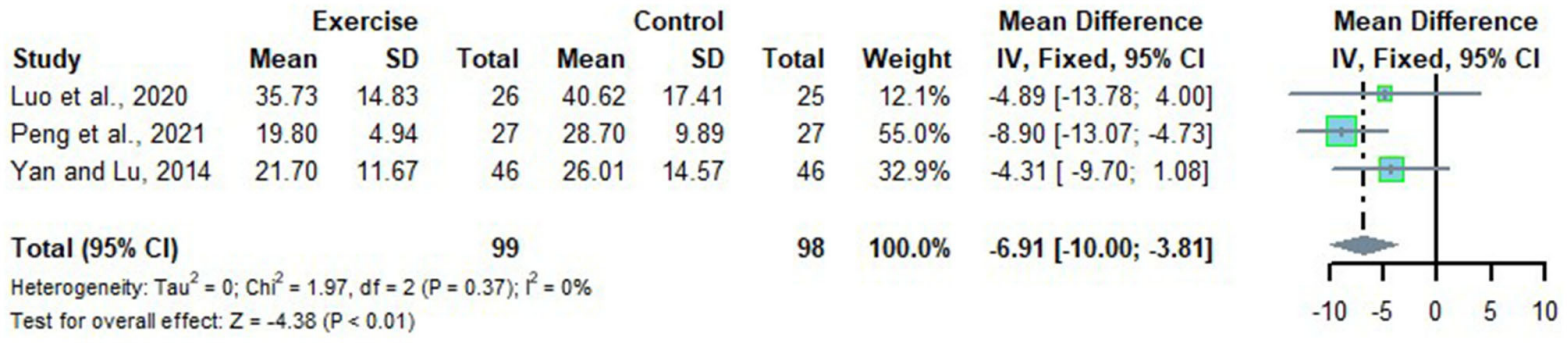
Meta-analysis of effects of long-term exercise on AST of Chinese patient with NAFLD.

Three studies looked at the alteration of AST in Chinese patients with NAFLD who received long-term exercise intervention with no heterogeneity detected (*I*^2^ = 0%, *p* = 0.37). Therefore, a fixed effects model was adopted for meta-analysis, and the results showed that the level of AST in Chinese patients with NAFLD was significantly reduced after long-term exercise intervention compared to that of the control group (*MD* = −6.91, 95%CI: −10.00 to −3.81, *p* < 0.01) (Figure 11).

## Discussion

In recent years, the risk of NAFLD in China has been increasing. Studies have confirmed the benefits of exercise for improving NAFLD, but there is no research focusing on the effect of long-term exercise on lipid metabolism in Chinese NAFLD patients. This study aims to explore the effect of long-term exercise on the lipid metabolism of Chinese NAFLD patients.

### Effects on BMI

Obesity is one of the important risk factors for NAFLD (Vernon et al., 2011). Therefore, obesity reduction can improve the status of NAFLD patients. Body mass index is widely used to identify individual obesity, and the BMI criteria for determining obesity in Chinese adults developed by the Chinese Obesity Working Group states that the reasonable range of BMI is 18.5–23.9 kg/m^2^ (Zhou, 2002). In the articles included in this study, the baseline of BMI in Chinese patients were all higher above this level. It shows that most Chinese patients with NAFLD are in obesity.

Our finding demonstrated that Chinese patients with NAFLD need to exercise longer than a 6 month to improve BMI significantly. But other studies held a different conclusion. For example, Zou et al. showed that exercise in any duration all had a beneficial effect on BMI in patients with NAFLD (Zou et al., 2018). Similar experimental results were also presented in Xiong's study (Xiong et al., 2021). The different results could be explained by the fact that the exercise intensity was not consistent across the literature included in the different studies. Higher intensity exercise can cause better fat loss in a short period, thus reducing BMI (Zhu et al., 2020). Most articles in our study used aerobic exercise as an intervention, and intensity was about 50–70% maximum heart rate, which did not fall within the intensity range of strenuous exercise. In contrast, in the studies by Zou et al. and Xiong et al., the character intensity of the long-term exercise intervention was less described. Therefore, lower exercise intensity may be why Chinese NAFLD patients need to exercise for longer periods to achieve improved BMI, and further studies are required in order to adjust the settings of long-term exercise intensity so that Chinese NAFLD patients can improve their BMI more efficiently.

### Effects on TC, LDL-C, and HDL-C

This systematic review and meta-analysis showed that long-term exercise could significantly improve TC of Chinese patients with NAFLD. It can be related to the high baseline level of TC in participants included in this study. According to guidelines for the prevention and treatment of dyslipidemia in Chinese adults (Zhu et al., 2016), a reasonable range for TC should not exceed 5.2 mmol/L. However, there are more than half of the participants higher than this level. In this condition, they are usually diagnosed as hyperlipidemia (Zhu et al., 2016). Other studies have been conducted to demonstrate the significant improvement effect of long-term exercise on TC in hyperlipidemia populations. In addition, the results of subgroup analysis showed that long-term exercise was more effective in improving TC in younger Chinese patients with NAFLD. The same results were also shown in another study (Costa et al., 2020). It may be related to differences in basal metabolic levels in humans at different ages.

The decrease in TC may also be associated with a reduction in LDL-C. Our study showed that long-term exercise significantly decreases LDL-C in Chinese patients with NAFLD, and LDL-C accounts for 20–30% of TC in the blood, so a decline in LDL-C is an important cause of TC decline.

There are two possible reasons for lower LDL-C. One is that long-term exercise may improve LDL receptor activity on hepatocyte membranes, enhance the liver's ability to transport LDL-C, thus reducing the level of LDL-C in the blood (Chen et al., 2005). Another possibility is that increased insulin sensitivity enhances the expression of LDL-R mRNAs, thereby accelerating the rate of LDL-C metabolism (Young and Stout, 1987). How exercise affects LDL-C is still unknown, but another study has demonstrated the effect of long-term exercise in improving LDL-C in patients with NAFLD (Fu et al., 2018). This study also reported the effect of long-term exercise on the improvement of HDL-C. Our study did not get the same conclusion. Through further analysis of the literature, we found that exercise intensity may be an important reason why there was no significant difference in HDL-C between exercise and control groups. In the article that get significant results, the intensity of the exercise selected was greater (Wang et al., 2020). Stein et al. concluded that the minimum intensity of improvement in HDL-C was 75% maximum heart rate, while in our included articles, exercise intensity was mostly maintained at 50–70% maximum heart rate. Therefore, it can be assumed that the reason for the failure of long-term exercise to increase HDL-C in Chinese patients with NAFLD is related to the selection of exercise intensity.

### Effects on TG

Our study shows that long-term exercise significantly reduces TG level in Chinese patients with NAFLD, which can be explained that long-term exercise increased lipolytic enzyme activity and accelerates the decomposition of TG into mitochondrial for energy supply (Boesch et al., 2010; Yu et al., 2017). In addition, the mechanism by which long-term exercise improves TG in Chinese patients with NAFLD may be related to adipocytokines such as leptin, adiponectin, and interleukin-6 (IL-6). Some studies have shown that adipocytokines are one of the causes of TG elevation (Izadi et al., 2013). The way in which long-term exercise actually improves adipokine levels still needs further research to confirm.

### Effects on ALT and AST

Alanine aminotransferase and aspartate aminotransferase are the most important transaminases. Elevated blood levels of ALT and AST are a sign that the liver is damaged. This systematic review and meta-analysis showed that long-term exercise significantly improves ALT and AST in Chinese patients with NAFLD. Similar results to ours have been reported in other studies (Smart et al., 2018; Ye et al., 2019). This could be considered the ability of long-term exercise to improve the inflammatory state of the liver by increasing SIRT activity and enhancing the deacetylation of key transcription factors of inflammation and metabolism, such as NF-κB and PGC-1α (Bianchi et al., 2021), which in turn improves the state of the liver. Thus, the changes may have benefited from the improvement in inflammatory state of liver.

## Strength and Limitations

This study is the first meta-analysis that focus on effect of long-term exercise on liver lipid metabolism in Chinese patients with NAFLD. Current evidence suggest that long-term exercise can improve almost all indicators related to lipid metabolism. This review searched a wide variety of databases including two Chinese electronic databases for relevant articles and two authors independently searched and selected the included studies, extracted the data, and assessed the risk of bias of every trial using recommended protocols and methodological schemes. Therefore, the results of this metaanalysis are considered a significant contribution.

However, this meta-analysis has several limitations. First, the quality of all the included trials in this meta-analysis was low. None of the studies detailed allocation concealment although all the trials used a randomization method. None of the studies met the blinding of participants though it seems unfeasible to use the blinding method in view of the exercise intervention. Second, the trials varied in methodological design (i.e., intervention methods) and conditions (i.e., duration). Thus, the outcomes have high heterogeneity although the total effects of outcomes were stable. Third, there may be some publication bias as unpublished articles could not be searched in this review although the funnel plot asymmetry did not show the bias.

## Conclusion

This systematic review and meta-analysis demonstrated that long-term exercise can significantly improve the levels of TG, TC, LDL-C, ALT, and AST in Chinese patients with NAFLD and exercise last more than 6 months can decrease BMI of Chinese patients with NAFLD.

## Data Availability Statement

The original contributions presented in the study are included in the article/Supplementary Material.

## Author Contributions

YG and JL designed the systematic review and supervised the entire program. YG and XL reviewed all the studies and extracted the information from the eligible trials. QM and JL analyzed the data and prepared the figures and table. YG, QM, and YS wrote the paper. YG, JL, and HS revised the manuscript. All authors reviewed and approved the manuscript.

## Funding

This work was supported by the National Key Research and Development Program of China (No. 2018YFC2000600) and Fok Ying Tung Education Foundation (No. 161094). The funders had no role in the study design, data collection and analysis, decision to publish, or preparation of the manuscript.

## Conflict of Interest

The authors declare that the research was conducted in the absence of any commercial or financial relationships that could be construed as a potential conflict of interest.

## Publisher's Note

All claims expressed in this article are solely those of the authors and do not necessarily represent those of their affiliated organizations, or those of the publisher, the editors and the reviewers. Any product that may be evaluated in this article, or claim that may be made by its manufacturer, is not guaranteed or endorsed by the publisher.
