# Supplementary material for: Effect of Long-Term Exercise on Liver Lipid Metabolism in Chinese Patients With NAFLD: A Systematic Review and Meta-Analysis
Source: Front Physiol. 2021 Nov 22;12:748517. doi: 10.3389/fphys.2021.748517 (PMC8646046; doi:10.3389/fphys.2021.748517)
Supplement: Supplementary file 1 [file Data_Sheet_1.docx]

Supplementary Material

**Search Strategies**

**PubMed**

#1 exercise [MeSH Terms]

#2 training [MeSH Terms]

#3 #1—#2/OR

#4 liver

#5 #3 AND #4

**Web of Science**

#1 TS= (exercise)

#2 TS= (training)

#3 #1—#2/OR

#4 TS= (liver)

#5 #3 AND #4

**China Science and Technology Journal Database (VIP)**

#1 U=运动

#2 U=训练

#3 U=锻炼

#4 #1—#3/OR

#5 U=肝脏

#6 #4 AND #5

**China National Knowledge Infrastructure (CNKI)**

#1 AB=运动

#2 AB=训练

#3 AB=锻炼

#4 #1—#3/OR

#5 AB=肝脏

#6 #4 AND #5
